# Effect of Glove Decontamination on Bacterial Contamination of Healthcare Personnel Hands

**DOI:** 10.1093/cid/ciz615

**Published:** 2019-09-13

**Authors:** Zegbeh Kpadeh-Rogers, Gwen L Robinson, Haleema Alserehi, Daniel J Morgan, Anthony D Harris, Natalia Blanco Herrera, Laura J Rose, Judith Noble-Wang, J Kristie Johnson, Surbhi Leekha

**Affiliations:** 1 Department of Pathology, Baltimore; 2 Department of Epidemiology and Public Health, University of Maryland School of Medicine, Baltimore; 3 Division of Healthcare Quality Promotion, National Center for Emerging and Zoonotic Infectious Diseases, Centers for Disease Control and Prevention, Atlanta, Georgia

**Keywords:** personal protective equipment, transmission, hand hygiene and gloves

## Abstract

We examined the effect of glove decontamination prior to removal on bacterial contamination of healthcare personnel hands in a laboratory simulation study. Glove decontamination reduced bacterial contamination of hands following removal. However, hand contamination still occurred with all decontamination methods, reinforcing the need for hand hygiene following glove removal.

## Teaser

In this laboratory simulation, we investigated the effect of glove decontamination prior to glove removal and found that glove decontamination reduced but did not eliminate bacterial contamination found on hands after glove removal.

Personal protective equipment (PPE), such as gloves and gowns, is used to protect healthcare personnel (HCP) from contamination by microorganisms. However, numerous studies have shown that HCP are at risk for self-contamination during the process of doffing contaminated PPE [1–6]. To reduce self-contamination during PPE removal, PPE decontamination prior to doffing was recommended by the Centers for Disease Control and Prevention (CDC) during the 2014–2016 Ebola outbreak [7]. Although theoretically beneficial, there are little empirical data to support PPE decontamination [8]. Further, while the consequences of self-contamination are apparent for a highly virulent pathogen such as Ebola, self-contamination during PPE removal may occur undetected for common bacterial and viral pathogens with the associated risk for transmission to subsequent patients, and therefore warrants further consideration. Our objective in this study was to identify the effect of glove decontamination on bacterial contamination of HCP hands following glove removal.

## METHODS

This simulation study, approved by the University of Maryland, Baltimore, Institutional Review Board, was performed in a controlled laboratory setting at the University of Maryland School of Medicine. HCP with direct patient care experience were enrolled as participants.

### Experimental Procedures

Experiments were conducted using methicillin-sensitive *Staphylococcus aureus* (MSSA) and an antibiotic-sensitive strain of *Klebsiella pneumoniae* as surrogates for methicillin-resistant *S. aureus* and carbapenem-resistant Enterobacteriaceae, respectively.

For each simulation, volunteers were asked to don 2 pairs of gloves and a gown, with the under gloves representing HCP hands and the top gloves representing the actual gloves worn for patient care. Simulation steps and sampling for bacterial load assessments were as follows: top gloves before decontamination, top gloves after decontamination of the top gloves with 1 of 3 products listed below, under gloves after top-glove removal without decontamination, and under gloves after top-glove decontamination with 1 of 3 products and top-glove removal.

For each experiment, the top gloves on both hands were directly inoculated with 50 µL of bacterial suspension and 50 µL of GloGerm Mist liquid fluorescent marker (GloGerm, Moab, UT) [6] to give a final concentration of 10^8^ colony forming units (CFU) of bacteria. A high inoculum was used based on our pilot observations that organism recovery from gloves was reduced by 1–2 logs from the original inoculum. Fluorescent marker was added to visually trace bacterial transfer throughout all experiments. Participants were asked to rub the bacteria/fluorescent marker on their hands in a standardized way. A research team member provided verbal instructions to ensure that doffing steps were performed per CDC protocols [7]. Alcohol-based hand rub, 63% alcohol (Steris Corp, Mentor, OH) and 2 US Environmental Protection Agency–registered hospital disinfectants, dispatch bleach disinfecting wipes (Clorox Healthcare, Oakland, CA) and Sani-Cloth AF3 quaternary ammonium (“quat”) disinfecting wipes (PDI Healthcare, Montvale, NJ), were used for decontamination. Volunteers were asked to decontaminate in a manner that ensured they covered all parts of the glove surface including between all fingers. Using a single pump of the alcohol-based hand rub, volunteers rubbed both gloved hands together, similar to routine hand hygiene in the hospital, until the gloves were completely dry. For wipe-based decontamination, the volunteer used a single wipe to decontaminate both gloves with continuous wiping for at least 1 minute. We ensured a total manufacturer-recommended dwell or contact time, that is, time for which the glove surface remained visibly wet, of 3 minutes for quat and 1 minute for bleach.

At the end of the experiment, gloves were sampled using a 3M sponge-stick with 10 mL neutralizing buffer (St. Paul, MN) in a standardized manner to ensure sampling of all surfaces. Sponge-sticks were processed using previously described methods [9]. From the eluent, successive 1/10 dilutions were made and plated on tryptic soy agar (Becton Dickinson, Sparks, MD) in triplicate for quantitative culturing. Plates were incubated overnight, and the number of CFUs of *K. pneumoniae* and MSSA were calculated. The eluent was also enriched in gram-negative broth (Becton Dickinson) for *K. pneumoniae* and tryptic soy broth with salt (Remel, Lenexa, KS) for MSSA, incubated overnight, and plated onto MacConkey agar and blood agar, respectively.

### Statistical Analyses

Reduction in bacterial load was assessed as follows: top-glove bacterial load reduction after decontamination compared with before decontamination (to ensure that disinfection was effective in reducing the contamination on the glove surface), bacterial load reduction after glove removal without a decontamination step (to evaluate the effect of wearing gloves in reducing contamination of hands), and bacterial load reduction after glove decontamination and removal (to evaluate the impact of glove decontamination on reducing bacterial load on hands beyond the effect of wearing gloves). The reduction in bacterial load after glove removal with decontamination compared with glove removal without decontamination was the primary outcome of interest. 

The Wilcoxon rank sum test was used for pairwise comparison of median CFUs between any 2 groups. Based on proposed methods for sample size calculations of the difference in medians of positively skewed outcomes [10], a sample size of 9 would enable us to detect a difference of at least 2-log CFUs between median CFUs among paired samples with 80% power at a significance level of 0.05. In addition to the quantitative differences, we also examined qualitative detection (presence/absence) of bacteria and fluorescent marker on the under glove after top-glove decontamination and glove removal.

## RESULTS

In total, 20 HCP (10 per organism) were enrolled. Of the 10^8^ CFU inoculated, the median recovery from top gloves (positive control) was 1.2 × 10^4^ CFU for both bacteria combined, 8.8 × 10^3^ CFU for MSSA, and 2.3 × 10^5^ CFU for *K. pneumoniae*. For both bacteria combined, the median bacterial recovery from the top glove after decontamination was 2.13 × 10^2^ CFU for alcohol and <10 CFU for quat and bleach. This translates to bacterial load reduction of 47%, or 1.75 log for alcohol, and >99%, or 4 log for quat and bleach; all *P* < .05.

### Contamination Reduction After Glove Removal Without Decontamination

After top-glove removal without any decontamination step, the median recovery from the under glove was 2.7 × 10^2^ CFU. Relative to the positive control, this translates to a bacterial load reduction of 98% (1.6 log) combined for both organisms (*P* < .01). By organism, the bacterial recovery was 1.7 × 10^2^ CFU for MSSA for a bacterial load reduction of 98% (1.7 log, *P* = .03) and 1.07 × 10^3^ CFU for *K. pneumoniae* for a bacterial load reduction of 99% (2.35 log, *P* = .08).

### Contamination Reduction After Glove Removal Following Glove Decontamination

**Figure 1.**
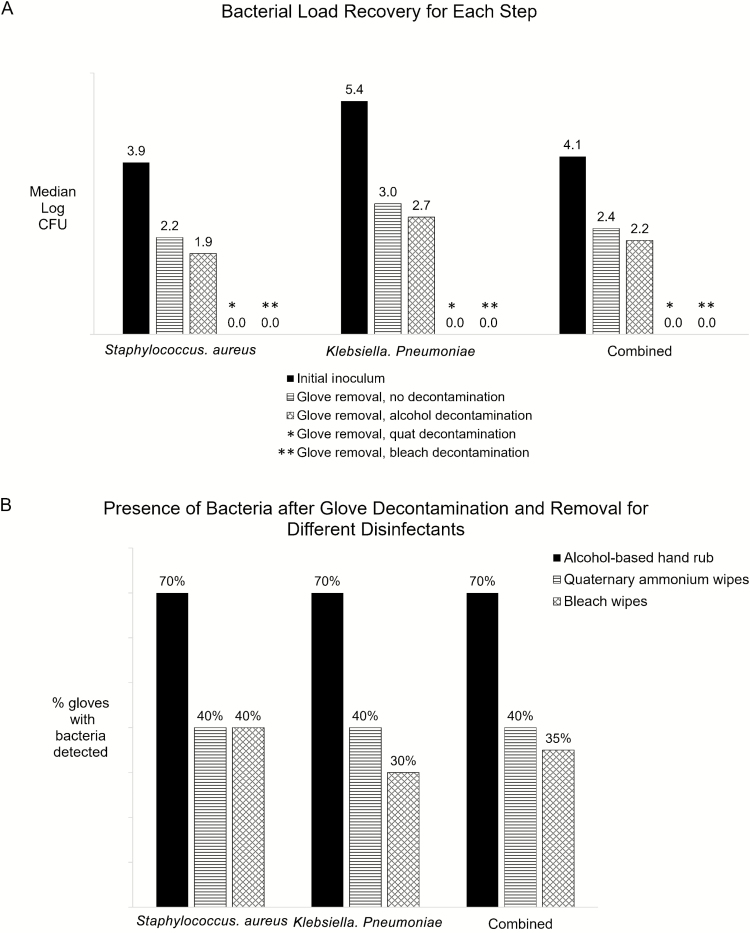
Effectiveness of glove decontamination. (*A*) Bacterial load of *Staphylococcus aureus* and *Klebsiella pneumoniae* for the steps of initial inoculation, glove removal without decontamination, and glove removal after decontamination with alcohol-based hand rub, quaternary ammonium wipes, and bleach wipes. (*B*) Bacterial qualitative detection after decontamination and glove removal. Abbreviation: CFU, colony-forming unit.

After top-glove decontamination and removal, the median bacterial recovery from the under glove was 1.4 × 10^2^ CFU for alcohol and <10 CFU for quat and bleach. This translates to bacterial load reductions of 47%, or 0.3 log for alcohol (*P* = .37) and 99%, or 2 log for both quat (*P* = .05) and bleach (*P* = .04; relative to bacterial load detected on under gloves after top-glove removal without decontamination). Reductions were similar for both organisms (Figure 1A).

### Fluorescent Marker and Qualitative Bacterial Detection

Measurable bacteria were recovered from under gloves even after top-glove decontamination and removal in 70%, 40%, and 35% of participants for alcohol, quat, and bleach, respectively. The fluorescent marker was recovered from the under gloves after top-glove decontamination and removal in 40%, 25%, and 35% of participants for alcohol, quat, and bleach, respectively, with similar findings for both MSSA and *K. pneumoniae* (Figure 1B).

## DISCUSSION

In this simulation study, we found that glove decontamination prior to glove removal reduces bacterial contamination of HCP hands. While we saw a significant quantitative reduction in bacteria with decontaminant use, bacteria could often still be detected after glove decontamination and removal. Findings were similar for both MSSA and *K. pneumoniae*. These results indicate that glove decontamination reduces but does not eliminate the risk of HCP self-contaminating their hands and potential transmission.

These results are consistent with the findings by Casanova et al who used the bacteriophage MS2 [11]. We also observed that bacterial reduction appeared to be greater for bleach and quaternary ammonium disinfectant wipes compared with alcohol-based hand rub. This may reflect either the physical removal of bacteria by the mechanical action of a wipe vs rubbing hands together or the effect of decontaminating agents. This finding needs confirmation.

The strengths of our study are the inclusion of HCP with direct patient care experience but not necessarily specialized training in doffing PPE and the use of bacteria (vs bacteriophage); these factors make the results applicable to routine patient care. A limitation of this study is that recovery of bacteria from gloved hands was much less (3–5 log reduction) than in a pilot study of methods to recover bacteria from gloves (1–2 log reduction; unpublished data). This could be due to differences in technique; in the pilot study, we inoculated and sampled only the palmar side of 1 gloved manikin hand. In the present study to mimic real-world scenarios, we asked HCP to rub and spread the inoculated bacteria on both hands, including the dorsum and in between fingers. All areas of the hand were sampled. Despite this low overall recovery of bacteria from gloves, we were able to demonstrate both a decrease in quantitative recovery after decontamination as well as residual contamination. We did not examine HCP hands as a potential source of MSSA but we ensured hand hygiene was performed prior to each experiment. Similar findings for MSSA and *K. pneumoniae* also support that the results represent inoculated MSSA vs endogenous MSSA colonizing HCP hands. Another potential explanation for the detection of bacteria on hands is penetration of bacteria through the glove material itself; however, this should be an infrequent occurrence with the medical-grade gloves used in our study and needs to be investigated in future work [12].

While the “dose,” or quantity, of bacteria on HCP hands resulting in subsequent transmission is unknown, reduction in bacterial load from glove decontamination, as found in this study, could potentially decrease transmission risk. Glove decontamination may be an adjunct technique for particularly virulent bacteria in the future. However, hand hygiene after glove removal remains important even in the setting of glove decontamination.

## Notes

***Financial support.*** This work was supported by the CDC Prevention Epicenters Program (grant 1U54CK000450-01 to Z. K. R., G. L. R., D. J. M., A. D. H., N. B. H., J. K. J., and S. L.).

***Supplement sponsorship.*** This article appears as part of the supplement “Personal Protective Equipment for Preventing Contact Transmission of Pathogens: Innovations from CDC’s Prevention Epicenters Program,” sponsored by the CDC’s Prevention Epicenters Program

***Potential conflicts of interest.*** D. J. M. reports grants from the CDC, National Institutes of Health, Agency for Healthcare Research and Quality, and U.S. Department of Veterans Affairs Health Services Research & Development; honoraria from Springer Nature, Inc; and other compensation from the Infectious Diseases Society of America and Society for Healthcare Epidemiology of America for expenses to organize or present at national meetings, outside the submitted work. All other authors report no potential conflicts of interest. All authors have submitted the ICMJE Form for Disclosure of Potential Conflicts of Interest. Conflicts that the editors consider relevant to the content of the manuscript have been disclosed.

## References

[1] Tomas ME, Kundrapu S, Thota P, et al. Contamination of health care personnel during removal of personal protective equipment. JAMA Intern Med 2015; 175:1904–10. 26457544 10.1001/jamainternmed.2015.4535

[2] Guo YP, Li Y, Wong PL Environment and body contamination: a comparison of two different removal methods in three types of personal protective clothing. Am J Infect Control 2014; 42:e39–45. 24679582 10.1016/j.ajic.2013.12.021 PMC7115291

[3] Casanova L, Alfano-Sobsey E, Rutala WA, Weber DJ, Sobsey M Virus transfer from personal protective equipment to healthcare employees’ skin and clothing. Emerg Infect Dis 2008; 14:1291–3. 18680659 10.3201/eid1408.080085 PMC2600382

[4] Casanova LM, Teal LJ, Sickbert-Bennett EE, et al; Centers for Disease Control and Prevention–Prevention Epicenters Program Assessment of self-contamination during removal of personal protective equipment for Ebola patient care. Infect Control Hosp Epidemiol 2016; 37:1156–61. 27477451 10.1017/ice.2016.169

[5] Zamora JE, Murdoch J, Simchison B, Day AG Contamination: a comparison of 2 personal protective systems. CMAJ 2006; 175:249–54. 16880444 10.1503/cmaj.060094 PMC1513425

[6] Kwon JH, Burnham CD, Reske KA, et al. Assessment of healthcare worker protocol deviations and self-contamination during personal protective equipment donning and doffing. Infect Control Hosp Epidemiol 2017; 38:1077–83. 28606192 10.1017/ice.2017.121 PMC6263164

[7] Centers for Disease Control and Prevention. Guidance on personal protective equipment to be used by healthcare workers during management of patients with Ebola virus disease in U.S. hospitals, including procedures for putting on (donning) and removing (doffing) Available at: http://www.cdc.gov/vhf/ebola/healthcare-us/ppe/guidance.html. Accessed 15 June 2018.

[8] Verbeek JH, Ijaz S, Mischke C, et al. Personal protective equipment for preventing highly infectious diseases due to exposure to contaminated body fluids in healthcare staff. Cochrane Database Syst Rev 2016; 4:CD011621. 27093058 10.1002/14651858.CD011621.pub2 PMC10068873

[9] Rose LJ, Hodges L, O’Connell H, Noble-Wang J National validation study of a cellulose sponge wipe-processing method for use after sampling *Bacillus anthracis* spores from surfaces. Appl Environ Microbiol 2011; 77:8355–9. 21965403 10.1128/AEM.05377-11 PMC3233038

[10] O’Keeffe AG, Ambler G, Barber JA Sample size calculations based on a difference in medians for positively skewed outcomes in health care studies. BMC Med Res Methodol 2017; 17:157. 29197347 10.1186/s12874-017-0426-1 PMC5712177

[11] Casanova LM, Erukunuakpor K, Kraft CS, et al; Centers for Disease Control and Prevention–Prevention Epicenters Program, Division of Healthcare Quality Promotion Assessing viral transfer during doffing of Ebola-level personal protective equipment in a biocontainment unit. Clin Infect Dis 2018; 66:945–9. 29471475 10.1093/cid/cix956 PMC6927896

[12] Wiwanitkit V Bloodborne viral pathogens and the feasibility of passing thorough the gloves: an appraisal and implication on infection control. Am J Infect Control 2006; 34:400. 16877113 10.1016/j.ajic.2006.02.001

